# Investigating practice integration of independent prescribing by community pharmacists using normalization process theory: a cross-sectional survey

**DOI:** 10.1007/s11096-024-01733-x

**Published:** 2024-05-10

**Authors:** L. Karim, T. McIntosh, T. Jebara, D. Pfleger, A. Osprey, S. Cunningham

**Affiliations:** 1 School of Pharmacy and Life Sciences, Robert Gordon University, Garthdee Road, Aberdeen, AB10 7GJ UK; 2 Health Services and Population Science Department, Institute of Psychiatry, Psychology and Neuroscience, De Crespigny Park, King’s College London, London, SE5 8AF UK; 3 Pharmacy and Medicines Directorate, Westholme, Woodend Hospital, Queens Road, Aberdeen, AB15 6LS UK; 4 Community Pharmacy Scotland, 42 Queen Street, Edinburgh, EH2 3NH UK

**Keywords:** Community pharmacy services, Implementation science, Non-medical prescribing, Pharmaceutical services, Systems theory

## Abstract

**Background:**

Independent prescribing (IP) has not been extensively investigated in community pharmacy (CP). Normalization process theory (NPT) constructs help explain how interventions are integrated into practice and include: ‘coherence’ (understanding), ‘cognitive participation’ (what promotes engagement), ‘collective action’ (integration with existing systems), and ‘reflexive monitoring’ (evaluation).

**Aim:**

To use NPT to investigate the integration of pharmacist IP in CP.

**Method:**

NHS Scotland Pharmacy First Plus (PFP) is a community pharmacy IP service. Questionnaire items were developed using the NPT derived Normalisation MeAsure Development (NoMAD) tool for an online survey of all PFP IP pharmacists. Demographic data were analysed descriptively and scale scores (calculated from item scores for the 4 NPT constructs) were used for inferential analysis.

**Results:**

There was a 73% (88/120) response rate. Greater than 90% ‘strongly agreed’/‘agreed’ to NoMAD items relating to most NPT constructs. However, responses to ‘collective action’ items were diverse with more participants answering ‘neither agree nor disagree’ or ‘disagree’. A statistically significant difference in NPT construct scale scores with significant *p*-values (ranging from *p* < 0.001 to *p* = 0.033) was shown on all the NPT constructs for the variable ‘On average, how often do you consult with patients under the PFP service?’.

**Conclusion:**

This theory-based work offers perspectives on IP integration within CP. Despite its geographic focus this work offers insights relevant to wider contexts on IP integration. It shows ‘collective action’ focused ‘organisation’ and ‘group process’ challenges with a need for further work on staff training, resource availability and utilisation, working relationships, communication and management.

**Supplementary Information:**

The online version contains supplementary material available at 10.1007/s11096-024-01733-x.

## Impact statements

- Independent prescribing (IP) by pharmacists exists in several countries and can impact positively patient services but its integration into community pharmacy (CP) has not been extensively investigated.
- The use of theory positively impacts the quality and relevance of pharmacy-based research and so this study uses the Normalization Process Theory (NPT).
- There is positivity to integration of IP in CP but a need for further consideration of aspects of the NPT ‘collective action’ construct ie. how IP integrates with existing systems and practices.
- Further work in this context is required on staff training, resource availability and utilisation, working relationships, communication and management.

## Introduction

Practice dimensions for health professionals are shifting with the integration of prescribing by non-medical health professionals. This includes pharmacists in the United Kingdom (UK), United States of America (USA), Canada and New Zealand [1–4]. Non-medical prescribing (NMP) has stated aims of improving patient care, patient safety and access to medicines and enhancing the utility of the skillset of health professionals [5–7].

Models of NMP practice are developing at differing rates and in differing ways around the world [8, 9]. The model that allows greatest flexibility for advancing patient care and professional practice is the independent prescribing (IP) model. In the UK, in 2006, regulations came into effect to allow pharmacists to prescribe independently [10] following successful completion of a certified training course [11]. In the USA, prescriptive authority using an IP ‘standard of care’ model has been implemented in a small number of states and is similar to the UK model of IP [2, 12]. In Canada, pharmacists have had IP rights for over 10 years [13] and the advantages of this model have been highlighted [14]. In New Zealand, it has been noted that there is variation in terms of regulation, educational programmes and prescribing competencies used by the different prescribing health professionals. The IP model is not yet available for pharmacists [15].

There is evidence relating to perceptions, views and attitudes towards IP in community pharmacy (CP) from Canada and the UK. This shows general enthusiasm and positivity, tempered with caution and forbearance [16–19] which can affect the integration of pharmacist prescribing into practice [20].

There is evidence that a range of factors at individual, organisational, regulatory and policy making levels influence the implementation of pharmacist IP in CP [18, 21]. In wider contexts, barriers to implementation of IPs have been summarised in a systematic review and are noted to exist at the ‘preparation’, ‘training’, ‘transition’ and ‘sustainment’ stages of implementation [22].

Given these challenges changing legislation and professional guidance is not sufficient to embed new practices [20]. Makowsky and colleagues used the ‘Diffusion of Innovations’ model in healthcare and showed a breadth of system-related factors influencing pharmacists taking on prescribing roles [23]. There is a need to extend the use of theory-based whole systems approaches to research in this area [24]. Robust research of CP services can be guided by theory-based implementation science approaches [25, 26].

**Fig. 1:**
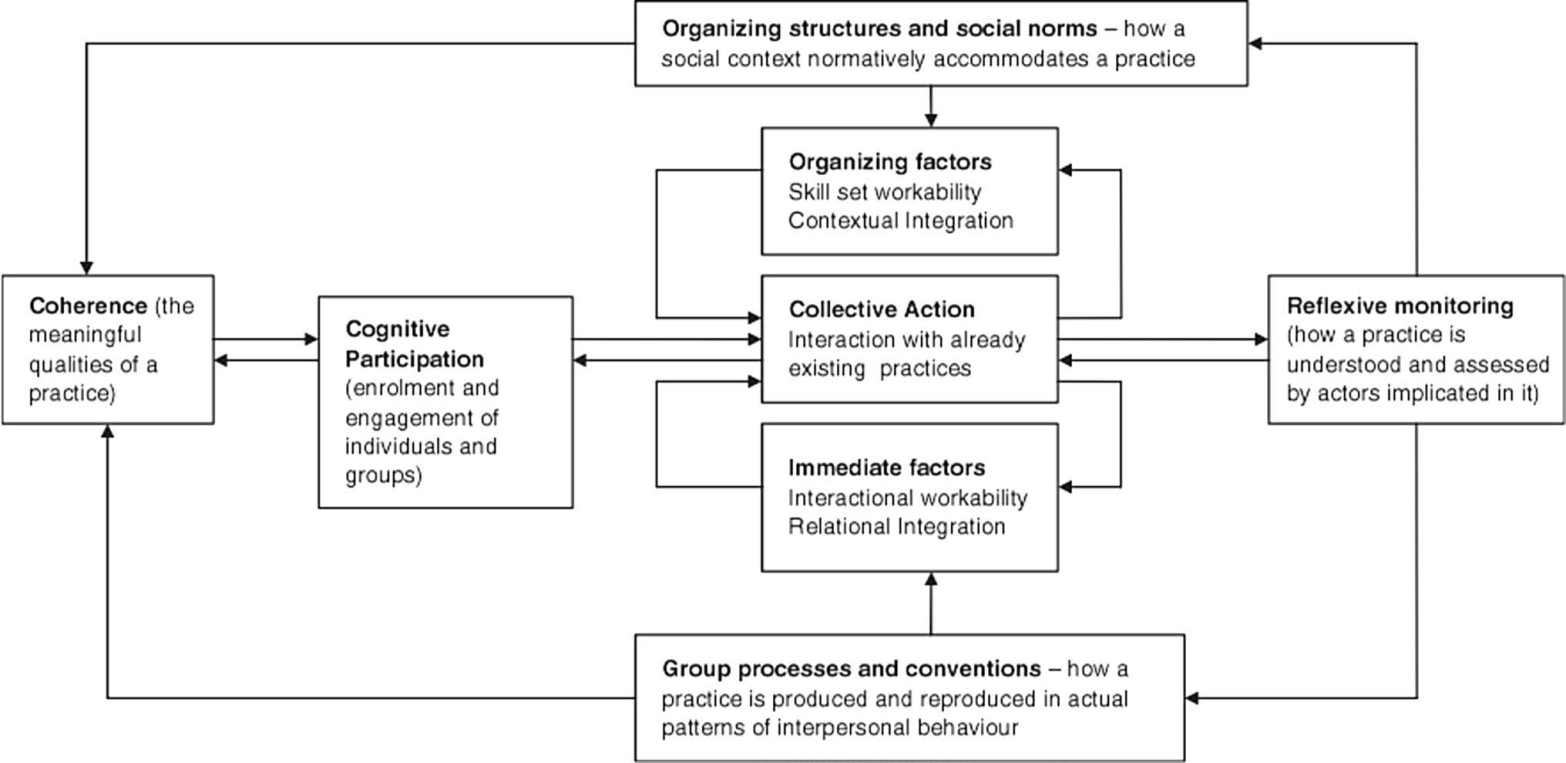
Normalization process theory (NPT)—an illustration of the components* NB: ‘Practice’ in the context of this research is ‘Pharmacist Independent Prescribing’ within community pharmacy. *Adapted from May C and Finch T. Implementing, Embedding, and Integrating Practices: An Outline of Normalization Process Theory. Sociology. 2009, 43(3): 535–554

One such approach is the normalization process theory (NPT) [27] which has four components (Fig. 1): 'coherence’ (meaningful qualities and understanding of benefits and possibilities of an intervention), ‘cognitive participation’ (what promotes enrolment in and engagement with an intervention), ‘collective action’ (how an intervention integrates with existing systems and practices), ‘reflexive monitoring’ (how integration of an intervention is evaluated and assessed). NPT is therefore designed to help explain how interventions are integrated (i.e. normalised) into practice and how the interventions work from early to later stages when embedded [28]. NPT was considered appropriate to use for this planned research in view of the need not simply to describe but consider relationships between factors influencing the implementation process at both individual practitioner and organisational levels and its use in this context has been advocated by other researchers [9].

In the UK, in November 2020, National Health Service (NHS) Scotland launched NHS Pharmacy First Plus (PFP) with the aim of supporting patients to access advice and treatment for common clinical conditions from pharmacist IPs in CP (within their competence and professional indemnity arrangements) rather than being referred to other healthcare professionals in other settings [29, 30]. There are no published research findings around this newly implemented initiative and this gap in evidence provides the rationale for this study.

### Aim

The aim of this work was to use NPT to investigate the integration of pharmacist IP in CP in the context of NHS Pharmacy First Plus.

### Ethics approval

Ethical approval (S307) was granted by Robert Gordon University, School of Pharmacy and Life Sciences on 2nd February 2022. The study was confirmed as exempt from full NHS ethical review by West of Scotland Research Ethics Service.

## Method

### Study design and setting

This cross-sectional online survey was carried out in CPs in Scotland in each of the 14 geographic Health Boards.

### Sample and sample size

At the time of the study (April–June 2022) a total of 120 CPs offering PFP were identified via NHS Board CP leads. All of these were invited to participate in the study with a request for an IP qualified pharmacist in each CP to complete the questionnaire. Given this the estimated population sample was 120 IP qualified pharmacists and using an online survey sample size calculator with: 95% confidence level, 120 population and 6% margin of error the ideal sample size is 83 [https://www.qualtrics.com/blog/calculating-sample-size/].

### Development of data collection tools

Demographic information on participants (Table 1) was collected. The Normalization MeAsure Development (NoMAD) items were used to develop the questionnaire for this study. NoMAD is a customisable tool based on NPT that is designed to capture aspects of intervention implementation into practices [31]. The items included (Tables 2 and 3): general questions related to perceptions of ‘familiarity’ and ‘’normality of the PFP service and items for each of the NPT constructs: coherence, cognitive participation, collective action and reflexive monitoring. Five-point semantic differential (‘not at all’ to ‘completely’) and Likert scales (‘strongly agree’ to ‘strongly disagree’) were used. A section for open comments was provided at the end of the questionnaire.

**Table 1:** Demographic data of questionnaire respondents (N = 88)

Demographic category	Number of respondents (%)
*Age*	
Less than 30 years	8 (9)
30–40 years	37 (42)
41–50 years	25 (28)
51–60 years	12 (13)
Greater than 60 years	6 (7)
*Gender*	
Male	31 (35)
Female	55 (63)
Would rather not say	2 (2)
Other	0
*Health Board of main practice setting*	
NHS Grampian	18 (21)
NHS Greater Glasgow and Clyde	16 (18)
NHS Fife	12 (14)
NHS Lothian	10 (11)
NHS Tayside	9 (10)
NHS Highland	6 (7)
NHS Lanarkshire	6 (7)
NHS Ayrshire and Arran	5 (6)
NHS Dumfries and Galloway	3 (3)
NHS Borders	2 (2)
NHS Forth Valley	1 (1)
NHS Western isles	0
NHS Orkney	0
NHS Shetland	0
*Employment category*	
Pharmacy manager	42 (48)
Pharmacist	28 (32)
Superintendent pharmacist	24 (27)
Other (including Locum, Pharmacy Owner Contractor, Area Manager, Pharmacy Owner, Area Manager)	8 (9)
*How many staff do you have working alongside you in your pharmacy when offering Pharmacy First Plus?*	
3 or fewer staff	30 (34)
4–6 staff	36 (41)
Over 6 staff	22 (25)

**Table 2:** General assessment responses for the Normalization MeAsure Development (NoMAD) questionnaire items relating to familiarity and normality (N = 88)

	Not at all	n (%)			Completely	Median (IQR)
Item	1	2	3	4	5	
When you deliver Pharmacy First Plus, how FAMILIAR does it feel to you?	4 (5)	11 (13)	29 (33)	26 (30)	18 (21)	3.5 (3 to 5)
To what extent do you feel Pharmacy First Plus is currently a NORMAL PART of your work?	4 (5)	8 (9)	22 (25)	27 (31)	27 (31)	4 (3 to 5)

**Table 3:** Response, internal consistency and scale scores data for Normalization MeAsure Development (NoMAD) items (N = 88)

NPT Construct	Statement	Strongly agree	Agree	Neither agree nor disagree	Disagree	Strongly disagree
		n (%)	n (%)	n (%)	n (%)	n (%)
Coherence	I am aware of how Pharmacy First Plus differs from usual ways of working in community pharmacy	50 (56.8)	35 (39.8)	1 (1.1)	2 (2.3	0
	Staff in this pharmacy have a shared understanding of the purpose of Pharmacy First Plus	41 (46.6)	39 (44.3)	4 (4.5)	4 (4.5)	0
	I understand how Pharmacy First Plus affects the nature of my own work e.g., my decision-making process/processes to undertake consultations etc	52 (59.1)	36 (40.9)	0	0	0
	I can see the potential value of Pharmacy First Plus for my role as a pharmacist independent prescriber	70 (79.5	16 (18.2)	2 (2.3)	0	0
	*Internal consistency: Cronbach's alpha 0.737 Scale score: Range 4 to 20, Midpoint 12. Median 19 (IQR 17–20)*					
Cognitive participation	There are key people in my organisation who drive Pharmacy First Plus forward	35 (39.8	28 (31.8)	19 (21.6)	3 (3.4)	3 (3.4)
	I believe that participating in Pharmacy First Plus is an integral part of my role	51 (58)	31 (35.2)	3 (3.4)	3 (3.4)	0
	I am open to working in new ways to effectively offer Pharmacy First Plus	61 (69.3)	25 (28.4)	2 (2.3)	0	0
	I intend to actively engage with Pharmacy First Plus when required	63 (71.6	25 (28.4)	0	0	0
	*Internal consistency: Cronbach's alpha 0.669 Scale score: Range 4–20, Midpoint 12, Median 18 (IQR 16–20)*					
Collective action	I can easily integrate Pharmacy First Plus into my current workflow in the pharmacy	18 (20.5	28 (31.8)	25 (28.4)	14 (15.9)	3 (3.4)
	Pharmacy First Plus disrupts working relationships within the pharmacy*	1 (1.1)	6 (6.8)	14 (15.9)	37 (42)	30 (34.1)
	I have confidence in other pharmacist's ability to offer Pharmacy First Plus	25 (28.4)	29 (33)	26 (29.5)	6 (6.8)	2 (2.3)
	Tasks are assigned to those with skills appropriate to Pharmacy First Plus	33 (37.5)	36 (40.9)	19 (21.6)	0	0
	Sufficient training is provided to staff	17 (19.3)	35 (39.8)	24 (27.3)	12 (13.6)	0
	Trained staff often offer Pharmacy First Plus to eligible patients	20 (22.7)	44 (50)	14 (15.9)	7 (8)	3 (3.4)
	Sufficient staff are available to support me in offering Pharmacy First Plus	15 (17)	31 (35.2)	19 (21.6)	18 (20.5)	5 (5.7)
	Management of the community pharmacy adequately supports Pharmacy First Plus	23 (26.1)	36 (40.9)	19 (21.6)	7 (8)	3 (3.4)
	*Internal consistency: Cronbach's alpha 0.680 Scale score: Range 8 to 40, Midpoint 24, Median 30 (IQR 26–33)*					
Reflexive monitoring	I have received feedback about the benefits of Pharmacy First Plus from my patients	39 (44.3)	38 (43.2)	10 (11.4)	1 (1.1)	0
	The staff within my pharmacy believe that Pharmacy First Plus is beneficial to our patients	45 (51.1)	38 (43.2)	5 (5.7)	0	0
	I value the effects that Pharmacy First Plus has had on my professional development	60 (68.2)	24 (27.3)	4 (4.5)	0	0
	I think feedback about Pharmacy First Plus can be used to improve it in the future	55 (62.5)	32 (36.4)	1 (1.1)	0	0
	I can modify how I deliver Pharmacy First Plus in response to feedback if necessary	50 (56.8)	37 (42)	1 (1.1)	0	0
	*Internal consistency: Cronbach's alpha 0.827 Scale score: Range 5 to 25, Midpoint 15, Median 23 (IQR 20–25)*					

*Item reversed scored

Face and content validity was tested using a sample of key IP and CP stakeholders in each Health Board across Scotland. Additionally, ‘Think Aloud’ testing [32] was carried out with three pharmacists with experience of pharmacy practice, education and academic research. This involved separate one-to-one online meetings with the lead researcher (LK). All aspects of data collection documentation were included i.e. the email invitation, instructions and questionnaire items. Each aspect was systematically considered, and the pharmacists were encouraged to verbalise their thoughts and understanding of each aspect and to enable the lead researcher to explore any areas of ambiguity and lack of clarity. Finally, the online questionnaire was piloted with five IPs who met the inclusion criteria to test the integrity and useability of the online systems. Pilot data were included in the final data set since no changes were made.

### Data collection

The questionnaire was hosted on the JISC Online Surveys (www.onlinesurveys.ac.uk). In April 2022 a link to the online questionnaire was sent via email by contacts at each regional Scottish Health Board to all community pharmacies across Scotland who offer PFP. Three reminder emails were sent at 2 weekly intervals and the survey was closed at the start of June 2022. A participant information sheet was provided and consent to participate was assumed through completion and submission of the questionnaire.

### Analysis

Data were exported to the IBM SPSS Statistics (SPSS Inc., Cary, NC version 21.0). Analysis was guided by the research aim and included descriptive and inferential statistics including Cronbach’s alpha scale item internal consistency testing (describing, with alpha values between 0 and 1, the extent to which the NPT construct scale score items are related to each other and so the construct, higher alpha values show higher internal consistency) and significance testing of scale scores with relevant nominal data using the Kruskal–Wallis test (considered statistically significant at a *p*-value of less than 0.05). The NoMAD related items were scored and analysed using the methods outlined by the original authors [31] with Likert scale items scored 5 for ‘strongly agree’ to 1 for ‘strongly disagree’. Open comments were analysed using framework analysis to identify key themes [33] and are presented in ‘Supplementary Materials’.

## Results

### Demographic data

The response rate was 88 of the 120 (73%) pharmacists who at the time of the study were providing PFP. Table 1 shows that the majority of respondents were female (63%, 55/88), under 40 years old (51%, 45/88), had worked in CP for more than 15 years (56%, 49/88) and had been qualified as an IP for less than 5 years (59%, 52/88).

Responses were received from pharmacists working in all the Health Board areas of Scotland where PFP was being provided. There was a greater number of responses (60%, 53/88) from the larger Health Board areas of Greater Glasgow and Clyde (population served 1.15 million), NHS Lothian (population served 0.8 million), NHS Grampian (population served 0.6 million) and NHS Tayside (population served 0.4 million). There was also strong representation from Health Boards with more rurality including NHS Highland, NHS Grampian, and NHS Tayside (38%, 33/88).

### NHS pharmacy first plus: activity and staffing levels

**Fig. 2:**
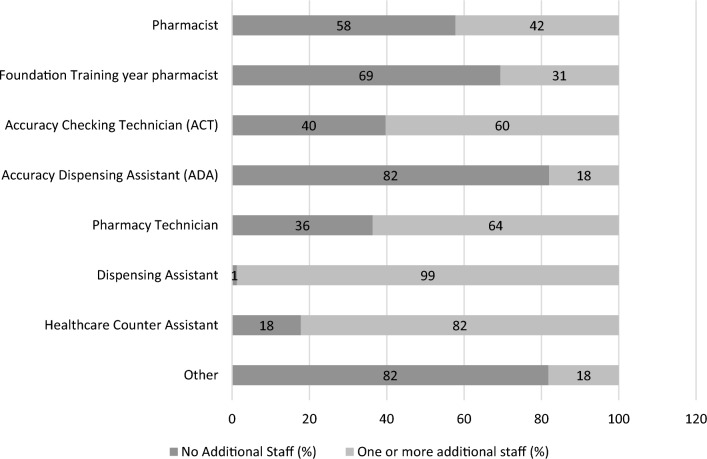
Additional staff working alongside responding independent prescribing pharmacists when providing NHS Pharmacy First Plus (N = 88, some missing data)

Table 1 shows that 76% (67/88) of respondents indicated that on average they consulted with patients under PFP service six or more times a week. The majority (66%, 58/88) indicated they had 4 or more staff working alongside them in their pharmacy when offering PFP. Figure 2 provides data on respondents’ reports of the characteristics of staff working alongside them on an average day when they were offering PFP. The majority (58%, 41/71) indicated that they provided the service while working as the only pharmacist in the CP. Sixty percent (44/73) had 1 or more accuracy checking technicians, 64% (42/66) one or more pharmacy technicians, 75% (60/80) had 2 or more dispensing assistants. Thirty-one percent (19/62) of respondents had a Foundation Training Year (formerly pre-registration) pharmacist.

### NoMAD (NPT) Questionnaire item responses

The NoMAD questionnaire items include ‘General Assessment’ questions that provide an indication of familiarity and how normal the respondent feels a service is in their working practice. Table 2 indicates that respondents were generally positive about ‘familiarity’ and ‘normality’ with medians of 3.5 (Inter-quartile range (IQR) 3–5) and 4 (IQR 3–5) respectively.

Table 3 provides data on the responses to each of the items devised to relate to the PFP service in line with the NPT constructs of ‘coherence’, ‘cognitive participation’, ‘collective actions’ and ‘reflexive monitoring’. Generally, there were high levels of agreement with more than 90% of the respondents ‘strongly agreeing’ or ‘agreeing’ to all items relating to ‘coherence’ and most relating to ‘cognitive participation’. One outlier was the item ‘There are key people in my organisation who drive PFP forward’ with only 53% (47/88) in agreement. Similarly, with ‘reflexive monitoring’ most items had greater than 90% in agreement with slightly fewer at 87% (77/88) in agreement with the item ‘I have received feedback about the benefits of PFP from my patients’.

Responses to the items within the ‘collective action’ construct were more diverse with a greater proportion of respondents answering ‘neither agree nor disagree’ or disagreeing.

An analysis of open comments provided by respondents indicates respondents’ willingness to adopt and integrate this new service into their already substantial workloads, and the urgent need to improve communication with GP practices including access to patient notes to facilitate this. Respondents expressed a need for additional training and staff resource with many working in a very demanding role as the sole pharmacist while providing the PFP service. A full analysis of comments with respondent quotations is provided in ‘Supplementary Materials’ provided alongside this article.

### Construct scale scores

As outlined above scale scores for each of the four NPT constructs were calculated through summation of item scores from within each construct for each respondent. Prior to this the items were tested for internal consistency (i.e. how well they related to each other) using Cronbach’s alpha. Cronbach’s alpha (a) for each of the four NPT construct groupings showed: ‘Coherence’ consisted of four items and α = 0.737; ‘cognitive participation’ had four items and α = 0.669); ‘collective action’ comprised eight items and α = 0.68; and ‘reflexive monitoring’ contained five items and α = 0.827. The normalisation scale overall (comprising items across all four constructs), was highly reliable (21 items, α = 0.852).

The scale scores for each respondent and construct were used to calculate range, midpoint and median responses (Table 3) and for further analysis. The generally positive nature of responses as outlined above is shown through consideration of the median scores and scale midpoint. The higher the median above the midpoint the more positive the responses to those items within the scale. The medians for ‘coherence’ and ‘reflexive monitoring’ were 7 and 8 points above the midpoint respectively. Those for ‘cognitive participation’ and collective action were 6 points above the midpoint. The greater diversity of responses to ‘collective action’ is shown by the larger inter quartile range (IQR) value of 7 compared to the IQR value for other constructs.

### Inferential statistics

Statistical testing showed no significant relationships between demographic characteristics (Table 1) and NoMAD NPT construct scale scores.

It was hypothesised that the participants professional experience and frequency of PFP consultation may have affected the responses to questionnaire items and so the NPT construct scale scores. The Kruskal–Wallis test was used to test for differences between NPT construct scale scores by calculating the ‘mean rank’ for each category within the professional experience and frequency of consultation variables (Table 4).

**Table 4:** Statistical relationships between NPT construct scale scores, participant professional experience and frequency of NHS Pharmacy First Plus consultation (N = 88)

Participant professional experience and frequency of consultation	n	Coherence	Cognitive Participation	Collective Action	Reflexive Monitoring
		Scale score mean rank*/ ‘*p* value’	Scale score mean rank/ ‘*p* value’	Scale score mean rank/ ‘*p* value’	Scale score mean rank/ ‘*p* value’
*How long qualified as pharmacist independent prescriber*					
less than 1 year	16	39.5	38.34	40.28	39.88
1–5 years	36	47.11	45.1	44.31	49.21
6–10 years	18	49.69	50.69	50.67	44.39
Greater than 10 years	18	38.53	42.58	42.47	39.31
		/*p* = 0.407	/*p* = 0.533	/*p* = 0.457	/*p* = 0.658
*How many years working in community pharmacy?*					
less than 1 year	0	0	0	0	0
1–2 years	0	0	0	0	0
3–5 years	3	45.50	17.33	27.00	36.33
6–10 years	20	53.88	48.10	48.88	50.33
11–15 years	16	46.19	51.13	46.31	45.63
Greater than 15 years	4	40.06	42.53	43.19	42.26
	9	/*p* = 0.209	/*p* = 0.147	/*p* = 0.608	/*p* = 0.529
*On average, how often do you consult with patients under the Pharmacy First Plus service?*					
Never	0	0	0	0	0
Fewer than 5 times per week	213	37.38	34.24	33.05	37.88
6–10 times per week	38	40.62	40.57	44.22	35.08
More than 10 times per week	29	54.74	57.09	53.16	61.64
		/*p* = 0.022	/*p* = 0.003	/*p* = 0.023	/*p* < 0.001

*Kruskal–Wallis used to test for differences between NPT construct scale scores mean ranks (and so median values) across ‘experience’ and ‘frequency of consultation’ variables

‘Mean rank’ values are similar across the variable categories and there were no statistically significant relationships between ‘How long qualified as pharmacist independent prescriber’ and ‘How many years working in community pharmacy?’ (Table 4).

The Kruskal–Wallis test revealed that there was a statistically significant relationship between frequency of PFP consultation activity and scale scores for all of the NPT constructs: ‘coherence’ (KW H 7.652, *p* = 0.022), ‘cognitive participation’ KW H 11.790, *p* = 0.033, ‘collective action’ (KW H 7.588, *p* = 0.023 and ‘reflexive monitoring’ (KW H 20.484, *p* = 0.001).

Higher ‘mean rank’ values for the category ‘More than 10 times per week’ for variable ‘On average, how often do you consult with patients under the Pharmacy First Plus service?’ indicates that those participants that undertook more PFP activity were more likely to agree to the items and so have positive views in relation to the NPT construct.

## Discussion

### Key findings

Respondents were generally positive about the service with high levels of agreement with all the items relating to the NPT constructs of ‘coherence’, ‘cognitive participation’ and ‘reflexive monitoring’. Responses to ‘collective action’ were more diverse with a greater proportion of respondents answering ‘neither agree nor disagree’ or disagreeing. A statistically significant difference in NPT construct scale scores with significant *p*-values (ranging from *p* < 0.001 to *p* = 0.033) was shown on all the NPT constructs for the variable ‘On average, how often do you consult with patients under the PFP service?’ with higher ‘mean rank’ values for ‘More than 10 times per week’.

### Strengths and limitations

The survey was sent to all community pharmacies in Scotland that at the time offered PFP with an excellent response from rural Heath Boards where PFP has an important role in improving access to healthcare [34]. The overall response rate resulted in a sample size that meets the 95% confidence level. A robust development process was undertaken using the previously validated NPT derived NoMAD tool and items were scored and analysed with reference to the methods outlined by the original authors [31, 35]. The Cronbach’s alpha calculated for the items included in each of the four NPT construct groupings showed high internal consistency.

Limitations include a proportionate excess from some NHS Board areas but in view of the response rate overall it was felt that it would not have been useful to follow up the non-responders. Notwithstanding that the sample size of 83 was achieved, the small available sample size means that statistical analysis may be under-powered and this may have led to no statistical difference findings.

### Interpretation

In relation to shared understanding of IP service provision in CP and so the NPT construct ‘coherence’ (Fig. 1), respondents indicated high levels of familiarity with the PFP service. The clear policy for and structure of contracted CP pharmacist prescribing services in Scotland may be facilitating this [29]. Makowsky and colleagues have highlighted this ‘innovation system fit’ facet as a significant factor in pharmacists adopting prescribing practices [23]. This work did not focus on service users’ understanding of community pharmacist prescribing services but it has been shown there is a need to raise service users’ awareness of such services [36].

Regarding the ‘cognitive participation’ construct (Fig. 1), the majority of respondents had been qualified for IP for less than 5 years. Faruquee and Guirguis concluded in their scoping review that increased risk and liability are demotivators for taking on a prescribing role and so activity is often higher in those with more experience and advanced qualifications [16]. The relatively recently-qualified participants in this study expressed willingness to engage with IP in the context of PFP, and these participants’ self-reported levels of prescribing shows that a possible lack of experience and advanced qualifications does not seem to have negative influence on IP integration.

Of relevance to the ‘collective action’ construct (Fig. 1) and specifically ‘organizing structures’, Edward and colleagues have synthesised the literature on barriers and facilitators to implementation of NMP in primary care in the UK [22] and identified the importance of organisational support for early adopters of prescribing practice. The findings from our work indicate potential organisational support barriers including a need for further consideration of: managerial/leadership support, challenges around interprofessional working, and communication including the availability and use of information communication technology (ICT) systems. The need for improvement in ICT in this context has recently been highlighted by others [37] along with the need for ICT evaluation frameworks [38]. ICT is also central to the ‘reflexive monitoring’ construct to allow the collation and analysis of prescribing data for audit and feedback purposes and so quality improvement of patient services.

The greater diversity of responses within the ‘group processes and norms’ aspect of ‘collective action’ (Fig. 1) indicates that there is a need for even greater clarity of team members roles, consideration of availability of training and funding for more staff resource and processes for working and communicating within teams. The influence of such factors on implementation of pharmacist prescribing has been shown by others in the primary care context [18, 21, 39].

This work was UK focused where there is a coherent NMP legislative and regulatory frameworks across the devolved nations, but implementation of IP is progressing at different rates and in different ways [29, 40]. This situation is reflected in the implementation of the models of IP for pharmacists in other countries including USA, Canada and New Zealand as outlined above [12, 13, 15]. Despite this, in an umbrella review [9] and other work [41, 42] have highlighted commonality internationally with respect to models and definitions, legal frameworks, outcomes and benefits, stakeholder satisfaction and barriers and facilitators to implementation. It is likely, therefore, that the results of this work will be applicable internationally.

### Further work

Further research could focus on defining the concepts and contexts relating to operationalisation of PFP and particularly the ‘collective action’ facets of the NPT. This in turn would help to ensure standardisation in relation to further evaluative studies on integration issues. Specific interventions could then be developed with cognisance of the Medical Research Council guidance on developing and evaluating complex interventions [43].

## Conclusion

This theory-based work offers a robust and unique perspective on IP integration within CP. The generally positive findings highlight challenges within the ‘collective action’ construct and a need to focus on training, staff resource, working relationships, communication and management. Despite the focus of this work it is likely that these factors are applicable to other jurisdictions and contexts.

### Supplementary Information

Below is the link to the electronic supplementary material.

Supplementary file1 (DOCX 20 kb)
