# Supplementary material for: Investigating practice integration of independent prescribing by community pharmacists using normalization process theory: a cross-sectional survey
Source: Int J Clin Pharm. 2024 May 10;46(4):966–76. doi: 10.1007/s11096-024-01733-x (PMC11286670; doi:10.1007/s11096-024-01733-x)
Supplement: Supplementary file 1 — Supplementary file1 (DOCX 20 kb) [file 11096_2024_1733_MOESM1_ESM.docx]

**Investigating practice integration of independent prescribing by community pharmacists using Normalization Process Theory: a cross-sectional survey**

**Karim, L^a^, McIntosh T^a^, Jebara T^b^, Pfleger D^c^, Osprey A^d^, Cunningham S^a^**

1. School of Pharmacy & Life Sciences, Robert Gordon University, Garthdee Road, Aberdeen, AB10 7GJ
2. Health Services & Population Science Department, Institute of Psychiatry, Psychology & Neuroscience, De Crespigny Park, King's College London, London, SE5 8AF
3. Pharmacy & Medicines Directorate, Westholme, Woodend Hospital, Queens Road, Aberdeen, AB15 6LS
4. Community Pharmacy Scotland, 42 Queen Street, Edinburgh, EH2 3NH

**Corresponding author:**

Professor Scott Cunningham

Professor of Pharmacy Education & Practice, School of Pharmacy & Life Sciences, Robert Gordon University, Garthdee Road, Aberdeen, AB10 7GJ

Email: [s.cunningham@rgu.ac.uk](mailto:s.cunningham@rgu.ac.uk)

**Supplementary Materials**

**S1. Content analysis of open comments**

The high levels of agreement in the three NoMAD constructs: ‘coherence’, ‘cognitive participation’ and ‘reflexive monitoring’ were in contrast to those in the ‘collective action’ construct, where there was less agreement and less positivity. Participants’ open comments in the questionnaire also reflected this.

Themes are highlighted in bold and illustrative quotations are provided with an indication of the respondent’s characteristics including: number of PFP consults per week, staff working alongside whilst offering PFP and years qualified as IP.

Integration of PFP into usual **working patterns was felt to be challenging**, with documentation and communication with GP practices particularly so.

“Documenting Pharmacy First Plus consultations takes time. This information then needs to be copied and sent to GP practice. They then scan into [the] patient’s file. IP community pharmacists need access to GP systems to cut down on admin and improve communication.” (>10 times per week, 4-6 staff, IP for 1-5 years)

Participants highlighted the **potential difficulties inherent in making clinical decisions without access to sufficient information**.

“The communicating each interaction with GP surgeries is the most time consuming part of this role and we sometimes feel we are acting in the dark as we don’t have access to notes.” (6-10 times per week, 4-6 staff, IP for 6-10 years)

Given that the service is offered across Scotland, one participant suggested the need for **provision of nationwide GP contact information**.

“I see a lot of tourists and there isn't even a list of GP practice clinical e-mails meaning we have to phone and get this before sending info through - this seems like something that could easily be pulled together to help make our lives easier!” (6-10 times per week, has 3 or fewer staff, IP for 6-10 years)

**Uptake of the service by patients varied** with some pharmacists spending a large proportion of their time in PFP consultations while others described much lower uptake.

“Community pharmacy is a very challenging place to work…..The stress of undertaking multiple consultations a day including required paperwork and consultation notes/SBARs and then to do the normal day’s work is very challenging. I feel over-worked on a daily basis with very little support from higher up management (despite raising concerns).” (fewer than 5 times per week under PFP, has 3 or fewer staff, IP for 1-5 years)

“Despite promoting within our social media sites, pharmacy app, local HSCP prescribing team and GP surgeries, uptake is surprisingly small. That said, we are so busy with our usual workload that it would be difficult to imagine how I would fit everything in if numbers were big.” (6-10 times per week, has 4-6 staff, IP for >10 years)

The **need for additional training** was highlighted.

“We have had to learn new skills on the job. As the programme evolves and evidence is gathered about conditions treated, hopefully adequate info and training programmes will be offered so we are adequately prepared.” (Fewer than 5 times per week, has 3 or fewer staff, IP for >10 years)

These comments describe respondents’ willingness to adopt and integrate this new service into their already substantial workloads and the urgent need to improve communication with GP practices including access to patient notes to facilitate this. Respondents expressed a need for additional training and staff resource with many working in a very demanding role as the sole pharmacist while providing the PFP service.

**S2. Further validation of NoMAD derived questionnaire items**

As outlined in the paper, scale scores for each of the four NPT constructs were calculated through summation of item scores from within each construct for each respondent. In order to assure the validity of taking this approach the items were tested for internal consistency (i.e. how well they related to each other) by calculation of Cronbach’s alpha.

Additionally, and in line with the procedures taken by the original developers of the NoMAD tool [1] the correlations between the construct measures scores and the overall normalisation score with the general assessment items are shown in Table A.

Correlations were generally low to moderate (r=0.279 to r=0.410). Of the two general assessment items relating to ‘familiarity’ and ‘normality’, the four construct measures appear to relate most strongly to perceptions that PFP has become a ‘familiar’ part of practice within CP with three of the four r values greater than 0.3.

**Table A. Bivariate correlation between NoMAD ‘General assessment’ questions and construct scale scores (N=88)**

|  |  | **Construct** | | | |
| --- | --- | --- | --- | --- | --- |
|  |  | **Coherence** | **Cognitive Participation** | **Collective Action** | **Reflexive Monitoring** |
| **NoMAD ‘General assessment’ questions** | |  |  |  |  |
| When you deliver Pharmacy First Plus, how FAMILIAR does it feel to you? | Pearson Correlation  (Sig - 2 tailed) | 0.297  (p=0.005) | 0.353  (p<0.001) | 0.302  (p=0.004) | 0.327  (p=0.002) |
| To what extent do you feel Pharmacy First Plus is currently a NORMAL PART of your work? | Pearson Correlation  (Sig - 2 tailed) | 0.279  (p=0.009) | 0.290  (p=0.006) | 0.410  (p<0.001) | 0.296  (p=0.005) |
| **NoMAD (NPT) construct scale scores** | |  |  |  |  |
| **Coherence** | Pearson Correlation  (Sig - 2 tailed) | 1 |  |  |  |
| **Cognitive Participation** | Pearson Correlation  (Sig - 2 tailed) | 0.607  (p<0.001) | 1 |  |  |
| **Collective action** | Pearson Correlation  (Sig - 2 tailed) | 0.581  (p<0.001) | 0.507  (p<0.001) | 1 |  |
| **Reflexive Monitoring** | Pearson Correlation  (Sig - 2 tailed) | 0.597  (p<0.001) | 0.672  (p<0.001) | 0.427  (p<0.001) | 1 |

Bivariate correlations between the NPT construct measures are shown in Table A. This shows moderate levels of correlation for summated scores within the construct domains as they relate to PFP integration into CP practice. This indicates that 'cognitive participation’ and 'reflexive monitoring’ are the most highly correlated (r=0.672), and ‘collective action’ and ‘reflexive monitoring’ (r=0.427) are the two constructs that are least correlated.

Supplementary Materials References:

1. Finch TL, Girling M, May CR et al. Improving the normalization of complex interventions: part 2 - validation of the NoMAD instrument for assessing implementation work based on normalization process theory (NPT). BMC Med Res Methodol. 2018;18:135.
